# Ensuring a good death: Priorities and concerns of healthcare personnel across healthcare settings

**DOI:** 10.1017/S1478951526102843

**Published:** 2026-06-18

**Authors:** Siren Eriksen, Anne Marie Mork Rokstad, Elisabeth Wiken Telenius, Kariann Krohne

**Affiliations:** 1 Norwegian National Centre on Ageing and Health, Vestfold Hospital Trust, Tønsberg, Norway; 2 Department of Postgraduate studies, Lovisenberg Diaconal University College, Oslo, Norway; 3 Faculty of Health Sciences and Social Care, Molde University College, Molde, Norway; 4 Department of Rehabilitation Science and Health Technology, Oslo Metropolitan University, Oslo, Norway; 5 Department of Public Health Science, Norwegian University of Life Sciences, Ås, Norway

**Keywords:** End-of-life care, palliative care, healthcare settings, healthcare personnel, focus group interviews

## Abstract

**Objectives:**

The primary objective of healthcare personnel involved in end-of-life care is to ensure a good and dignified death. However, end-of-life care is delivered across various healthcare settings. This qualitative study aimed to identify the priorities and concerns related to end-of-life care shared by healthcare personnel working in hospitals, nursing homes, and municipal home care.

**Methods:**

Twenty-two healthcare personnel from across Norway participated in virtual focus group interviews. Participants represented 3 distinct healthcare settings and had diverse professional backgrounds. Each interview was audio-recorded and transcribed verbatim. Qualitative content analysis was conducted and informed by theory on professional competence.

**Results:**

The analysis demonstrated that ensuring a good death was a shared focus among participants. Across settings, they expressed aligned priorities and concerns regarding end-of-life care. They likened their role to that of a pilot boat skipper, guiding the end-of-life process with a clear focus on: 1) offering support and information, 2) providing symptom relief, and 3) ensuring a presence at the bedside. Each of these themes highlights a key priority in end-of-life care across healthcare settings, along with its associated concerns.

**Significance of results:**

This study demonstrates that healthcare personnel across hospitals, nursing homes, and municipal home care share core priorities of what constitutes a good and dignified death. The findings challenge setting-specific assumptions in end-of-life care and support an emphasis on shared core competencies in education and practice. Overall, the study contributes to a more unified understanding of end-of-life care by highlighting foundational care values that transcend organizational boundaries and professional backgrounds, thereby supporting policies that promote continuity and coherence across care environments.

## Introduction

While some individuals die unexpectedly, the majority follow a predictable process that necessitates end-of-life care. Consequently, end-of-life care becomes a crucial professional competency for most healthcare personnel. The primary objective in end-of-life care is to facilitate a *good death* and prevent a *bad death*. Research literature identifies several core elements of a good death from the perspective of healthcare personnel. A central aspect is not dying alone (Gjerberg and Bjørndal 2007; Bratcher 2010), but rather dying peacefully (Beckstrand et al. 2006; Ko et al. 2015; Bovero et al. 2020) in the presence of close family and/or friends (Borgstrom 2024). Additionally, effective management of pain and other distressing symptoms, coupled with the avoidance of unnecessary life-prolonging interventions, is frequently cited as a fundamental component of a good death (Gjerberg and Bjørndal 2007; Bratcher 2010; Ko et al. 2015; Krikorian et al. 2020; Borgstrom 2024).

In contrast, Ko et al., drawing on interviews, describe a bad death as: “Experiencing death by accident or violence, prolonging life with life support; becoming dependent while entering a dying trajectory; and dying alone” (2015, 425). In their scoping review, Wilson and Hewitt (2018) found that between 7.8% and 23% of all deaths were considered bad deaths. Among the contributing factors, unrelieved pain was most frequently identified as associated with bad deaths. The study also identified organizational factors, including delayed initiation of end-of-life care and interpersonal or team conflicts. Beckstrand et al.’s survey (2006) highlighted organizational challenges to a peaceful end-of-life experience, including limited staff time, communication barriers, and treatment decisions driven more by personnel preferences than patient needs. The latter may suggest that normative expectations or ideals of a good death are linked to end-of-life care.

In Norway, 50% of deaths occur in nursing homes, 30% in hospitals, and 15% at home (Kalseth and Theisen 2017). Norwegian national health policies (Directorate of Health 2018; Ministry of Health and Care Services 2020) affirm the right of individuals with life-threatening illnesses to die with dignity, in accordance with their own expectations and values. These policies also emphasize that individuals in the final phase of life should be cared for by competent personnel who can provide medical treatment and ensure a dignified and safe end-of-life experience. Thus, the professional competence of healthcare personnel is explicitly highlighted. Professional competence refers to healthcare personnel’s ability to respond effectively to the specific demands presented within a given context (Nygren 2004). The 2 key dimensions of professional competence are relational competence, which involves building and maintaining communicative and emotional relationships with patients and families, and action competence, which pertains to the professional’s instrumental skills and knowledge. In clinical practice, these 2 dimensions are intertwined, as relational approaches are often necessary to achieve action-based goals (Nygren 2004).

While previous qualitative studies have examined the end-of-life care experiences of nurses (Bratcher 2010; Rahnama et al. 2023) and physicians (Fasting et al. 2022), or focused on end-of-life care in specific settings such as patients’ homes (Danielsen et al. 2018), hospitals (Di Leo et al. 2019), or nursing homes (Gjerberg and Bjørndal 2007), none have yet identified the common elements of end-of-life care across these healthcare settings. This insight is crucial for advancing care delivery, fostering collaboration, and ultimately improving the quality of life for patients with end-of-life care needs and their families. Supported by theory on professional competence (Nygren 2004), the aim of this study was to provide insight into the shared priorities and concerns of healthcare personnel working in different settings when organizing end-of-life care.

## Methods

This qualitative study adopted a social constructivist approach, using focus groups to encourage open dialogue among participants from the same employment setting. Focus groups allow participants to exchange and challenge viewpoints, fostering a deeper understanding of the topic (Morgan 1997; Krueger and Casey 2015).

### Recruitment and participants

Eligibility criteria included experience with end-of-life care and Norwegian language proficiency. Email invitations were sent to relevant units in 8 hospitals and 20 municipalities across Norway, via leaders who could forward them to eligible staff. Recruitment was further broadened through professional networks and targeted social media posts. Twenty-two individuals contacted the research team; none were excluded. The sample, comprising 2 men and 20 women, included 15 nurses, 3 physicians, 1 healthcare assistant, 1 physiotherapist, 1 chaplain, and 1 intellectual disability nurse. They had from 2 to 30 years of experience, and 8 had advanced palliative care training. The chaplain was employed in a healthcare institution and was formally recognized as healthcare personnel (Health Personnel Act 1999).

### Data collection

Five focus groups were organized according to employment setting (Table 1), with male participants in separate groups. The focus groups were conducted via Microsoft Teams in June 2022. They lasted from 84 to 93 minutes (mean, 88 minutes) and were audio-recorded using a Dictaphone. EWT and KK facilitated the nursing home and municipal home care groups, while an external researcher facilitated the hospital groups; all moderators were supported by external assistants. Moderators and assistants were trained in the focus group method, unfamiliar with participants, and maintained minimal involvement to encourage open discussion.

**Table 1.** Employment setting and professional backgrounds of focus group participants Table 1 long description.

Employment setting	Focus group number	Participants’ professional backgrounds
Nursing home	1	1 chaplain, 2 nurses, 1 healthcare assistant, 1 physiotherapist
Nursing home	2	1 intellectual disability nurse, 4 nurses
Municipal home care	1	4 nurses
Hospital	1	2 nurses, 2 physicians
Hospital	2	3 nurses, 1 physician

An interview guide based on the study aim and current literature was used. For this paper, the primary question of interest was: *What is your most important contribution as healthcare personnel when a patient has reached the final days of their life?* To ensure relevance across healthcare settings, including nursing homes, discussions focused on expected deaths among adults. Data collection continued until no substantially new information emerged.

### Data analysis

Recordings were professionally transcribed and analyzed using Graneheim and Lundman’s (2004) qualitative content analysis. In Step 1, all authors read the transcripts several times to gain an overview and agree on units of meaning. In Step 2, meaning units were extracted, condensed, and assigned codes. In Step 3, codes were compared, similarities and differences were identified, and a theme structure was established. In Step 4, themes were formulated into descriptive results, and quotations were coded by participant number, profession, setting, and group number. KK conducted Steps 2 and 3, supported by the other authors who equally reviewed preliminary codes and categories and engaged in regular interpretative team discussions. Any disagreements were resolved through discussion until consensus was achieved. All authors contributed to refining and validating the final themes in Step 4. The authors’ backgrounds in nursing, physiotherapy, and social anthropology supported critical reflection and theme refinement. Theory on professional competence (Nygren 2004) was used to contextualize the analysis and focus on how participants described required knowledge, skills, and contextual understanding (Braun and Clarke 2024). Thus, theory informed the interpretation as an analytical lens, without serving as a template or a set of predefined categories (Malterud 2016).

### Research ethics

The Norwegian Agency for Shared Services in Education and Research approved this study (no. 260146). Participants were given oral and written information about the study. All agreed not to share patient details or discuss content outside the group. Written informed consent was obtained.

## Results

The data analysis revealed the following themes: 1) offering support and information, 2) providing symptom relief, and 3) ensuring a presence at the bedside. Each theme highlights a main priority in end-of-life care across the settings, along with its associated concerns.

### Offering support and information

Participants described how they worked to make the dying process as good as possible for the patient and their family. Establishing an open dialogue and providing support were described as key priorities. Participants stressed the need to be available, approachable, and compassionate: “I think the most important thing for us is to be close and be able to talk and speak the same language – so that we understand each other” (P1, Chaplain, Nursing home 1). They summed their main role up as being a pilot boat skipper, guiding all involved through the process.

Participants noted that they sometimes knew the dying patient well, while at other times their first encounter occurred when the patient was already nonresponsive. In both situations, establishing a good relationship with the family was seen as essential because families provided valuable insights into the patient’s preferences. Participants also emphasized supporting families during the vigil, noting that the terminal phase could last for days or weeks and could be emotionally demanding. To help, they offered practical respite, such as a place to rest, a meal, a shower, or time outdoors.

They described preparing families for potentially distressing symptoms or sudden decline, but noted that stressed family members often struggled to absorb and retain information. To ease this distress, participants used various communication strategies, such as repeating information or introducing it gradually. They also described using a nonconfrontational communication strategy to ease into difficult topics:

One communication strategy that we use frequently, and that I feel encourages (further dialogue) is saying something like: “In our experience, many people in situations like yours, they feel … this and that. Can you relate to that?” (P2, Nurse, Hospital 1)

Furthermore, participants found it helpful to provide the family with a designated contact person, ideally an experienced palliative care professional, who could listen, explain, and help organize information from the different professionals involved in the patient’s care.

An underlying challenge evident in the participants’ accounts was that all end-of-life pathways are unique, requiring them to be prepared for unpredictable events. Lengthy care trajectories and sudden deaths added tension and stress:

The common obituary phrase, “died peacefully with close family present,” conveys an idealized image that often fails to reflect the realities encountered by those of us working in end-of-life care. We often see restlessness, anxiety, a lot of unresolved matters, need for control, and families in disarray. (P3, Nurse, Hospital 2)

Participants described making deliberate efforts to ease tensions, such as avoiding taking sides in family conflicts or helping to mediate them.

A man had fallen out with his sister many years ago. When he was dying … we were able to facilitate a visit from his sister. They had a conversation alone. I don’t know what they talked about, but he was extremely relieved after the conversation and needed noticeably less pain relief. (P1, Physician, Hospital 1)

Participants felt these experiences strengthened their catalytic role and their understanding of reconciliation’s impact.

### Providing symptom relief

Participants described adequate pain relief as essential to making the final days as comfortable as possible. They emphasized that good pain management also helped calm patients and reduce anxiety, noting that anxiety, distress, and restlessness in the terminal phase could worsen respiratory symptoms. They further explained that witnessing patient anxiety was distressing for families, and that reducing it improved both the family’s vigil experience and their perception of the staff’s end-of-life competence:

I believe that a crucial aspect of our work lies in our ability to effectively alleviate patients’ suffering and that the families witness our commitment to ensuring the best for the patient. When we succeed, it generates positive feedback; the family feels that their concerns are addressed because we’ve cared for their loved ones. (P2, Nurse, Nursing home 2)

Participants, across all groups, expressed concern that many dying patients – despite good intentions – were still receiving insufficient pain management.

We see this often, the patient has not been given enough, let’s say morphine. It says so time and time again (in the journal): (The pain relief had) little effect. But nothing happens … many are afraid to increase doses and give enough to provide good relief. (P4, Nurse, Nursing home 2)

They described how some colleagues harbored concerns about administering excessive pain medication, inadvertently leading to the patient’s death. Consequently, dosages of pain medication sometimes erred on the side of being too conservative. This practice was repeatedly described as unsatisfactory.

Participants recognized timely and adequate medical pain relief, including initiating, maintaining, and adjusting treatment, as a key clinical skill. Several described this as something that develops through experience and confidence, supported by access to knowledgeable colleagues and formal palliative care training: *The nurses often work alone, and if they don’t feel confident in titrating pain medication, they need someone to talk to. (…) So, they’ll often ring me up when changing the dosage* (P4, Nurse, Municipal home care 1).

Participants pointed out that pain often extends beyond a biological response. Across all focus groups, the participants discussed the occurrence of spiritual and existential pain. When such needs were expressed by patients or families, participants stated that they would gently offer to contact someone with expertise in spiritual and existential needs, such as a priest or imam, acknowledging their own limitations in providing spiritual, religious, or existential support.

### Ensuring a presence at the bedside

All participants affirmed that, unless a patient specifically requested to be alone, they made efforts to ensure someone was present with the dying patient. The value of having close family present was repeatedly emphasized, with comments such as “It is very important to have loved ones around, when you are at the end of your life” (P2, Nurse, Hospital 2) and “Nobody should die alone” (P2, Nurse, Nursing home 2) appearing across all groups.

Although ensuring a presence at the bedside was a common goal, participants acknowledged that not all patients had close family, not all wished for company, and not all families were able or willing to sit vigil at the time of death. In such cases, participants described how they themselves attempted to meet the patient’s need for human presence.

When we observe that death is imminent, and that they are all alone … when they have no family, no relatives around … then we’re there. We choose to be there because we don’t want them to die alone. (P3, Nurse, Nursing home 2)

They explained that, in practice, they visited the patients more frequently, spent longer periods with them, or arranged continuous presence through a sitting service.

Participants emphasized that bedside presence was not only physical but also emotional, requiring connection and awareness of the moment. For example, participants reported that during the COVID-19 pandemic, face masks and infection control clothing reduced their ability to connect with patients and contribute to a calming environment. Thus, in an effort to establish an emotional presence during COVID-19, several participants admitted to briefly removing their masks upon entering the patient’s room, to show the patient their face, while others, particularly staff from municipal home care, chose not to wear masks at all if the patient was not infected with COVID-19: “Simply because they were dying. They’re going to leave this world soon anyway, and we didn’t want the last thing they saw to be someone wearing a visor and a mask” (P3, Nurse, Municipal home care 1).

Participants believed that the physical and emotional presence of family contributed to a positive atmosphere. When relatives gathered at the bedside and held the patient’s hand, staff could step back and let the process unfold without unnecessary interruptions. However, participants explained that they were vigilant regarding family members who failed to provide what they saw as adequate emotional presence:

A woman was dying, and her husband was called. He came. (…) When I came into the room, he asked for help with the TV remote, because he wanted to watch some sports channel … I was a little surprised. He turned on the sports channel and sat down. And then maybe an hour goes by (…) and she dies. He was still watching TV …. (P1, Nurse, Hospital 2)

This participant later reflected on the possibility that the husband’s behavior might reflect their life together. When family ties were characterized by indifference or unrest, participants often reported reinforcing their emotional presence to support the patient. For example, they might sit with the family and speak to the dying patient on the relative’s behalf: “Now, Marie, your daughter is sitting here by your side … and the lilacs you love so much are blooming. They are here on your nightstand” (P2, Physiotherapist, Nursing home 1). This quote illustrates an effort to compensate for the family member’s lack of emotional presence.

## Discussion

In this study, we aimed to provide insights into the shared priorities and concerns of healthcare personnel across settings in organizing end-of-life care. Participants emphasized that their main priorities were offering support and information, providing symptom relief, and ensuring a human presence at the deathbed. Each priority was accompanied by practical and clinical concerns. The priorities and concerns identified in our study align with findings from previous research on healthcare personnel’s experiences in specific healthcare settings (Sekse et al. 2018; Quigley and McCleskey 2021; Peeler et al. 2023) and could therefore appear familiar. However, the consensus observed in this study suggests that these are central aspects of end-of-life care that transcend specific settings and populations.

Information sharing is central to end-of-life care, and consistent with prior research (Seccareccia et al. 2015; Tarberg et al. 2022), participants reported actively using nonconfrontational communication strategies. Transparent and truthful information sharing, open communication, and efforts to help families comprehend the end-of-life situation have been shown to enhance family support (Saarinen et al. 2023). Healthcare personnel are key providers of informational support, and they are well positioned to serve as families’ main contact person, addressing questions and concerns (Rahnama et al. 2023). Vermorgen et al. (2021) emphasized that families are both part of the palliative care team and individuals with their own care needs. Their interview study revealed shortcomings in communication between healthcare personnel and families, highlighting the need for a designated care coordinator or contact person. Our findings likewise underscored the importance of having such a contact person. In a systematic review, Mulcahy Symmons et al. (2023) found that collaboration between healthcare personnel and families is shaped by communication quality, family dynamics, and families’ awareness of patient preferences. Although these factors may be associated with relational conflicts, engaging in open discussions about the patient’s future care, as proposed by our participants, can help facilitate consensus between both parties.

Our findings illustrate how participants provide psychological, social, and spiritual support to patients and families in end-of-life care, respecting their preferences and wishes. These findings highlight the dual relational focus of end-of-life care, as participants’ work involves both the dying patient and their family. The participants recognized that families play an indispensable role, as they often share a unique bond with the patient that involves both personal dedication and valuable insights into their personality and care preferences. This has also been described in other studies (Carlsen and Lundberg 2018; Quigley and McCleskey 2021; Hou et al. 2022; Rahnama et al. 2023). A good relationship is believed to strengthen the family’s capacity to be present throughout the end-of-life process. As such, it is an investment and vital to providing high-quality end-of-life care (Tarberg et al. 2022).

Symptom relief and pain management are well-established cornerstones of end-of-life care and were strongly emphasized in our study. Pain significantly affects patients’ well-being, and the importance of adequate pain relief in end-of-life care is widely documented in international literature (Martinez et al. 2014; Smyth et al. 2018). Furthermore, participants discussed the undesirable practice of underdosing pain medication due to fears of overdosing. They stressed that the administration of adequate medical pain relief depends on the administrators’ professional competence and access to support from competent colleagues. Similar concerns have been reported in previous studies (Heneka et al. 2018, 2019).

For the participants, ensuring human presence at the patient’s bedside was a major priority. The presence of family was considered the most valuable. When family or others were unable to keep vigil, healthcare personnel endeavored to meet the dying patient’s need for human presence. A prominent finding in this study is that participants were concerned with the quality of the bedside presence, emphasizing that merely being physically present was not sufficient. The ideal presence was not perceived as a continuous physical presence, but rather as a continuity of attention. Through relational approaches, such as removing masks and visors during the COVID-19 pandemic to reveal their faces to patients or being particularly attentive to family members who were perceived as passive, they negotiated a shared emotional presence. While strong social relationships and emotional interconnectedness are known to contribute to a good death (Ko et al. 2015), the ways in which participants intervened and compensated for passive or emotionally absent family members have, to our knowledge, not been previously documented. Their approaches find support in a qualitative study by Aghaei et al. (2020), who argue that healthcare personnel may perceive the presence of family as insufficient to fully support the patient emotionally. At the same time, such relational approaches reveal healthcare personnel’s normative expectations regarding bedside presence. Normative expectations in end-of-life care are closely tied to the ideal of a good death. However, such expectations may reinforce paternalistic tendencies in care and unintentionally impede patient-centered care (Philip and Komesaroff 2006; Cottrell and Duggleby 2016; Callaghan and Fanning 2018).

Participants metaphorically compared their role to that of a pilot boat skipper navigating the complexities of end-of-life care. To fulfill this role, they described drawing on both *relational competence* and *action competence* in end-of-life care practice (Nygren 2004). The significance of action competence was most evident in symptom management, particularly in the provision of pain relief. However, communication also emerged as an area where action competence intersected with relational competence, as participants employed various strategies to address sensitive issues. Our findings further illustrate how participants relied on relational competence when providing psychological, social, and spiritual support for patients and family members, and when encouraging human bedside presence. Thus, being a pilot boat skipper navigating the waters of end-of-life care requires a high level of expertise, encompassing both action competence and relational competence, regardless of professional background and healthcare setting.

### Strengths and limitations

The cross-setting design is a key strength of the study. Focus groups were conducted using the same interview guide, supporting consistency while allowing for comparison across settings. The analysis involved multiple researchers and was conducted reflexively and transparently, with researchers’ preunderstandings explicitly acknowledged and critically examined through ongoing team discussions. That said, the study has several limitations to consider. First, nurses were the majority, which likely reflects Norwegian staffing patterns. Second, only 4 participants were recruited from municipal home care services; however, this should be understood in light of Norway’s low home death rates (Kalseth and Theisen 2017), which may influence both the availability and experience of personnel in this setting. Third, the use of multidisciplinary focus groups requires methodological consideration (Clavering and McLaughlin 2007). Although probing questions were used to include perspectives from all professions and minimize hierarchical influence, such dynamics may still have shaped the discussions. Another methodological consideration concerns the fact that the virtual format of the study may have influenced group interaction (Chai et al. 2024). To mitigate this, small group sizes and trained moderators were used. Participants were also guided to take part in a distraction-free environment.

### Implications for future practice

Palliative education and end-of-life training should be interprofessional and applicable across healthcare settings. Training should combine symptom-management competence with strong relational and communication skills and include ethical reflection, family support, and confidence in pain management. Participants’ accounts draw attention to the relational aspects of care and to normative assumptions about families’ emotional presence that are often overlooked in practice. Thus, educational programs should foster critical awareness of normative assumptions about what constitutes a “good death.”

Shared priorities across settings highlight the potential for stronger intersectoral collaboration, including common competence frameworks and structured communication pathways. Such collaboration can enhance continuity and reduce fragmentation during transitions.

## Conclusion

Clear communication, symptom relief, and bedside presence were consistent priorities across settings and professions, indicating core elements of end-of-life care relevant for education and practice. These findings underscore the importance of interprofessional education that integrates symptom management with communication skills, ethical reflection, and family-support strategies, and strengthens healthcare personnel’s use of both relational and action-based competencies in practice. Services should ensure continuity, clear contact points, and organizational structures that enable relational care, supported by access to experienced colleagues in complex situations. Further research is needed on the relational dynamics of bedside presence, particularly when family members struggle to provide emotional support.

## Data Availability

Data supporting this study’s findings are available from the corresponding author upon reasonable request.

## Table 1 Long description

The table lists five focus groups by employment setting and summarizes participants’ professional backgrounds in each group. Two groups are from nursing homes: one includes a chaplain, two nurses, a healthcare assistant, and a physiotherapist, while the other includes an intellectual disability nurse and four nurses. One municipal home care group includes four nurses only. Two hospital groups include both nurses and physicians: one has two nurses and two physicians, and the other has three nurses and one physician. Nurses appear in every group and are the largest professional group overall, while physicians appear only in hospital groups. The nursing home setting shows the greatest variety of roles compared with municipal home care and hospital groups.

Navigate back to Table 1.

## References

[ref1] Aghaei MH, Vanaki Z and Mohammadi E (2020) Emotional bond: The nature of relationship in palliative care for cancer patients. *Indian Journal of Palliative Care* 26(1), 86–94. doi:10.4103/ijpc.Ijpc_181_19 32132791 PMC7017707

[ref2] Beckstrand RL, Callister LC and Kirchhoff KT (2006) Providing a “good death”: Critical care nurses’ suggestions for improving end-of-life care. *American Journal of Critical Care* 15(1), 38–45 doi:10.4037/ajcc2006.15.1.38 16391313

[ref3] Borgstrom E (2024) What is a good death? A critical discourse policy analysis. *BMJ Supportive and Palliative Care* 14(e3), e2546–e2553. doi:10.1136/bmjspcare-2019-002173 32631959

[ref4] Bovero A, Gottardo F, Botto R, et al. (2020) Definition of a good death, attitudes toward death, and feelings of interconnectedness among people taking care of terminally ill patients with cancer: An exploratory study. *American Journal of Hospice and Palliative Care* 37(5), 343–349. doi:10.1177/1049909119883835 31648531

[ref5] Bratcher JR (2010) How do critical care nurses define a “good death” in the intensive care unit? *Critical Care Nursing Quarterly* 33(1), 87–99. doi:10.1097/CNQ.0b013e3181c8e2d7 20019515

[ref6] Braun V and Clarke V (2024) Reporting guidelines for qualitative research: A values-based approach. *Qualitative Research in Psychology* 22, 1–40. doi:10.1080/14780887.2024.2382244

[ref7] Callaghan KA and Fanning JB (2018) Managing bias in palliative care: Professional hazards in goals of care discussions at the end of life. *American Journal of Hospice and Palliative Care* 35(2), 355–363. doi:10.1177/1049909117707486 28486834

[ref8] Carlsen B and Lundberg K (2018) ‘If it weren’t for me…’: Perspectives of family carers of older people receiving professional care. *Scandinavian Journal of Caring Sciences* 32(1), 213–221. doi:10.1111/scs.12450 28475236

[ref9] Chai CA, Barrios M, Gómez-benito J, et al. (2024) Information retrieval in face-to-face and online focus groups. a systematic review. *International Journal of Qualitative Methods* 23, 16094069241286856. doi:10.1177/16094069241286856

[ref10] Clavering EK and McLaughlin J (2007) Crossing multidisciplinary divides: Exploring professional hierarchies and boundaries in focus groups. *Qualitative Health Research* 17(3), 400–410. doi:10.1177/1049732306298380 17301348

[ref11] Cottrell L and Duggleby W (2016) The “good death”: An integrative literature review. *Palliative & Supportive Care* 14(6), 686–712. doi:10.1017/S1478951515001285 26732508

[ref12] Danielsen BV, Sand AM, Rosland JH, et al. (2018) Experiences and challenges of home care nurses and general practitioners in home-based palliative care - a qualitative study. *BMC Palliative Care* 17(1), 95. doi:10.1186/s12904-018-0350-0 30021583 PMC6052702

[ref13] Di Leo S, Alquati S, Autelitano C, et al. (2019) Palliative care in the emergency department as seen by providers and users: A qualitative study. *Scandinavian Journal of Trauma, Resuscitation and Emergency Medicine* 27(1), 88. doi:10.1186/s13049-019-0662-y 31533807 PMC6751856

[ref14] Directorate of Health (2018) Lindrende behandling i livets sluttfase - Nasjonale faglige råd (Palliative care in end-of-life - National guidelines). https://www.helsedirektoratet.no/faglige-rad/lindrende-behandling-i-livets-sluttfase (accessed 25 April 2025).

[ref15] Fasting A, Hetlevik I and Mjolstad BP (2022) Finding their place - general practitioners’ experiences with palliative care - a Norwegian qualitative study. *BMC Palliative Care* 21(1), 126. doi:10.1186/s12904-022-01015-1 35820894 PMC9277777

[ref16] Gjerberg E and Bjørndal A (2007) Hva er en god død i sykehjem? (What is a good death in nursing homes?). *Sykepleien Forskning* 2(3), 174–180. doi:10.4220/sykepleienf.2007.0057

[ref17] Graneheim UH and Lundman B (2004) Qualitative content analysis in nursing research: Concepts, procedures and measures to achieve trustworthiness. *Nurse Education Today* 24(2), 105–112. doi:10.1016/j.nedt.2003.10.001 14769454

[ref18] Health Personnel Act (1999) Lov om helsepersonell m.v. (Act relating to Health Personnel etc). www.lovdata.no (accessed 22 April 2025).

[ref19] Heneka N, Bhattarai P, Shaw T, et al. (2019) Clinicians’ perceptions of opioid error-contributing factors in inpatient palliative care services: A qualitative study. *Palliative Medicine* 33(4), 430–444. doi:10.1177/0269216319832799 30819045

[ref20] Heneka N, Shaw T, Rowett D, et al. (2018) Opioid errors in inpatient palliative care services: A retrospective review. *BMJ Supportive & Palliative Care* 8(2), 175–179. doi:10.1136/bmjspcare-2017-001417 29307863

[ref21] Hou X, Lu Y, Yang H, et al. (2022) Preferences for a good death: A cross-sectional survey in advanced cancer patients. *BMJ Supportive & Palliative Care* 12(e4), e570–e577. doi:10.1136/bmjspcare-2018-001750 30944121

[ref22] Kalseth J and Theisen OM (2017) Trends in place of death: The role of demographic and epidemiological shifts in end-of-life care policy. *Palliative Medicine* 31(10), 964–974. doi:10.1177/0269216317691259 28190375

[ref23] Ko E, Kwak J and Nelson-becker H (2015) What constitutes a good and bad death? Perspectives of homeless older adults. *Death Studies* 39(7), 422–432. doi:10.1080/07481187.2014.958629 25674672

[ref24] Krikorian A, Maldonado C and Pastrana T (2020) Patient’s perspectives on the notion of a good death: A systematic review of the literature. *Journal of Pain and Symptom Management* 59(1), 152–164. doi:10.1016/j.jpainsymman.2019.07.033 31404643

[ref25] Krueger RA and Casey MA (2015) *Focus Groups: A Practical Guide for Applied Research*. Los Angeles, CA: SAGE Publisher.

[ref26] Malterud K (2016) Theory and interpretation in qualitative studies from general practice: Why and how? *Scandinavian Journal of Public Health* 44(2), 120–129. doi:10.1177/1403494815621181 26647095

[ref27] Martinez KA, Aslakson RA, Wilson RF, et al. (2014) A systematic review of health care interventions for pain in patients with advanced cancer. *American Journal of Hospice and Palliative Care* 31(1), 79–86. doi:10.1177/1049909113476129 23408371 PMC4711357

[ref28] Ministry of Health and Care Services (2020) Meld. St. 24 (2019–2020) Lindrende behandling og omsorg. Vi skal alle dø en dag. Men alle andre dager skal vi leve. (Report No. 24 to the Storting (2019-2020): palliative Care and Treatment. “We shall all die one day. But all other days we shall live”). https://www.regjeringen.no/no/dokumenter/meld.-st.-24-20192020/id2700942/?ch=1 (accessed 20 April 2025).

[ref29] Morgan DL (1997) *Focus Groups as Qualitative Research*. Thousand Oaks, CA: SAGE Publications.

[ref30] Mulcahy Symmons S, Ryan K, Aoun SM, et al. (2023) Decision-making in palliative care: Patient and family caregiver concordance and discordance-systematic review and narrative synthesis. *BMJ Supportive & Palliative Care* 13(4), 374–385. doi:10.1136/bmjspcare-2022-003525 PMC10804031 35318213

[ref31] Nygren P (2004) *Handlingskompetanse: Om Profesjonelle Personer (Action Competence: On Professional Persons)*. Oslo: Gyldendal akademisk.

[ref32] Peeler A, Doran A, Winter-dean L, et al. (2023) Public health palliative care interventions that enable communities to support people who are dying and their carers: A scoping review of studies that assess person-centered outcomes. *Frontiers in Public Health* 11. doi:10.3389/fpubh.2023.1180571 PMC10410270 37564426

[ref33] Philip JAM and Komesaroff P (2006) Ideals and compromises in palliative care. *Journal of Palliative Medicine* 9(6), 1339–1347. doi:10.1089/jpm.2006.9.1339 17187542

[ref34] Quigley DD and McCleskey SG (2021) Improving care experiences for patients and caregivers at end of life: A systematic review. *American Journal of Hospice and Palliative Care* 38(1), 84–93. doi:10.1177/1049909120931468 32551966 PMC8526304

[ref35] Rahnama M, Abdollahimohammad A, Asadi-bidmeshki E, et al. (2023) Nurses’ caring experiences for dying patients: A meta-synthesis review. *OMEGA - Journal of Death and Dying*, 302228231206513. doi:10.1177/00302228231206513 37837313

[ref36] Saarinen J, Mishina K, Soikkeli-jalonen A, et al. (2023) Family members’ participation in palliative inpatient care: An integrative review. *Scandinavian Journal of Caring Sciences* 37(4), 897–908. doi:10.1111/scs.13062 34958141

[ref37] Seccareccia D, Wentlandt K, Kevork N, et al. (2015) Communication and quality of care on palliative care units: A qualitative study. *Journal of Palliative Medicine* 18(9), 758–764. doi:10.1089/jpm.2014.0408 26069934

[ref38] Sekse RJT, Hunskår I and Ellingsen S (2018) The nurse’s role in palliative care: A qualitative meta-synthesis. *Journal of Clinical Nursing* 27(1-2), e21–e38. doi:10.1111/jocn.13912 28695651

[ref39] Smyth JA, Dempster M, Warwick I, et al. (2018) A systematic review of the patient- and carer-related factors affecting the experience of pain for advanced cancer patients cared for at home. *Journal of Pain and Symptom Management* 55(2), 496–507. doi:10.1016/j.jpainsymman.2017.08.012 28843458

[ref40] Tarberg AS, Thronaes M, Landstad BJ, et al. (2022) Physicians’ perceptions of patient participation and the involvement of family caregivers in the palliative care pathway. *Health Expectations* 25(4), 1945–1953. doi:10.1111/hex.13551 35765248 PMC9327811

[ref41] Vermorgen M, Vandenbogaerde I, Van Audenhove C, et al. (2021) Are family carers part of the care team providing end-of-life care? A qualitative interview study on the collaboration between family and professional carers. *Palliative Medicine* 35(1), 109–119. doi:10.1177/0269216320954342 32928056

[ref42] Wilson DM and Hewitt JA (2018) A scoping research literature review to assess the state of existing evidence on the “bad” death. *Palliative & Supportive Care* 16(1), 90–106. doi:10.1017/S1478951517000530 28655363

